# Cavitation dynamics and thermodynamic effect of R134a refrigerant in a Venturi tube

**DOI:** 10.1016/j.ultsonch.2024.107202

**Published:** 2024-12-15

**Authors:** Beile Zhang, Ze Zhang, Xufeng Fang, Rong Xue, Shuangtao Chen, Yu Hou

**Affiliations:** a School of Energy and Power Engineering, Xi’an Jiaotong University, Xi’an 710049, China; b MOE Key Laboratory of Cryogenic Technology and Equipment, Xi’an Jiaotong University, Xi’an 710049, China; c R&D Center, Dongfang Electric Machinery Co., Ltd, Deyang 618000, China

**Keywords:** Cavitating flow, Thermodynamic effect, R134a refrigerant, Venturi tube

## Abstract

Cavitation plays a crucial role in the reliability of components in refrigeration systems. The properties of refrigerants change significantly with temperature, thereby amplifying the impact of thermodynamic effects. This study, based on the Large Eddy Simulation (LES) method and the Schnerr-Sauer (S-S) cavitation model, investigates the transient cavitating flow characteristics of the R134a refrigerant in a Venturi tube (VT). The bubble number density in the S-S model was improved based on the experimental data of pressure and temperature. Simulation results indicate that there are two shedding modes of cavitation clouds in R134a refrigerant. One is induced by the combined action of reentrant flow and the vortices centrifugal force, while the other is generated by the central jet of the mainstream and the reverse jet produced by the collapsing cavitation bubbles. Furthermore, the thermodynamic effects of the refrigerant exert a certain inhibitory effect on cavitation, revealing the causes of instability in the refrigerant cavitation interface and the shedding characteristics of cavitation clouds. The relationship between local sound speed, flow velocity, and heat conduction rate in the cavitation region was studied, unveiling a time-lag in temperature changes relative to pressure changes in the intensive cavitation region. This study provides insights into the complex cavitation dynamics, especially in R134a refrigerant systems, and provides an approach for accurately predicting and managing cavitation in various industrial applications.

## 1 Introduction

Cavitation refers to the process of vapor bubble formation in low-pressure regions of the liquid flow field. [1] It is a hydrodynamics issue peculiar to turbomachinery such as pump [2], pump-turbines [3], and propellers [4], which use liquid as fluid medium. When pressure returns above the saturation level, cavities or cavitation bubbles begin to collapse, generating shock waves and cavitating jets. [5] Although cavitation, as an environmentally friendly processing technology, has found widespread application in fields such as wastewater treatment [6], [7], geological energy extraction [8], food processing and chemical engineering [9], [10], it can significantly impair the hydraulic performance of hydromachinery. The collapse of cavitation bubbles causes material surface damage and induces vibrations and noise, leading to irreversible damage to the stable and safe operation of the equipment. [11], [12].

In the study of hydrodynamic cavitation, non-rotational reactors, which are typically more cost-effective, are commonly used. [13] Due to the complex three-dimensional structure of hydromachinery, researchers typically decompose the impeller flow passages into simpler geometric structures such as VTs, hydrofoils and blunt bodies. To date, extensive numerical simulations and experimental studies have been conducted to investigate cavitating flow structures and the intricate cavitation dynamics. [14], [15] The accuracy of computational models is fundamental to simulation studies. Ghahramani *et al.* proposed a hybrid cavitation model capable of accurately capturing large and small-scale cavity structures. [16] Li *et al.* employed a LES methodology, integrating the volume of fluid approach with adaptive mesh refinement, to elucidate the phase intricacies and shedding dynamics characteristic of sheet-to-cloud cavitation. This computational framework is adept at capturing the morphological transitions of micro cavities in response to the propagation of pressure waves or condensation shock events. [17] Wang *et al.* introduced a quasi-one-dimensional theoretical model to characterize the dynamics of cavitation, and investigated the impact of liquid viscosity on the structure and collapse process of bubbles during cavitation, using a combined approach of experimentation and simulation. [18] Ji *et al.* conducted advanced investigations into cavitating flow and vortex distributions around a twisted hydrofoil, utilizing an enhanced RNG k-ε model in conjunction with a mass transfer cavitation model. Their findings indicate that vortices, induced by cavitation, facilitate fluid separation along boundary layers and contribute to increased flow instability. [19] To further enhance simulation accuracy and computational efficiency, Wang *et al.* employed the method of adaptive mesh refinement to examine the structures of cavities and the cavitating flow surrounding hydrofoils. Their study revealed that the velocity of cavitation cloud detachment and disintegration surpasses the expansion rate of affixed cavities. [20] Additionally, Ji *et al.* proposed a multi-scale Eulerian–Lagrangian method to capture macroscopic vapor structures and track microbubble behavior. Using a coupled two-way algorithm, they conducted a series of studies on the effects of cavitation vortices and turbulence on bubble dynamics. [21] Their analysis examined the formation mechanisms of cloud cavitation and the characteristics of cavity shedding, confirming the reliability of this method in predicting bubble evolution. [22], [23] Contrasting with the approach of Ji *et al.*, [19] Meng *et al.* utilized the boundary data immersion method combined with implicit LES to simulate the vortex structures and dynamics of cavitating flow. Their research revealed that the cavitation regions exhibit amplified turbulence and enstrophy, particularly influenced by velocity factors and reentrant jets. [24] Zhang *et al.* conducted research on the shedding mechanisms of cavitation clouds. They observed the unsteady cavitation shedding phenomena induced by reentrant jet, condensation shock wave, and collapse-induced pressure wave under different cavitation numbers and confirmed the distinctions among these three mechanisms. [25] Beyond the focus on cavitation behaviors surrounding hydrofoils, researchers have expanded their investigation to include the phenomena of cavitating flow within VTs. [26] Brunhart *et al.*’s CFD results indicate that the bubble shedding mechanism in VTs under different operating conditions can be categorized into two types: reentrant jet and condensation shock. [27] Hasani Malekshah *et al.* employed spectral analysis and grayscale image post-processing to scrutinize the evolution and transient properties of cavitating flow in VTs under the influence of dissolved gases, focusing on bubble morphology. Their findings revealed that the presence of dissolved air decelerates the cavity flow velocity, thus modifying the inception cavitation points and the progression of bubble formation. [28] Following this, they applied a Merging theory-modified cavitation model to investigate the effects of dissolved air on cavitation dynamics, noting that variations in cavitation numbers and air injection rates substantially impact the pressure coefficient, shedding frequency, and structural attributes of cavitation. [29].

In cavitation, the liquid vaporization persistently draws energy from the adjacent fluid, resulting in the formation of a temperature gradient between the cavitation region and its surrounding liquid. This occurrence, termed the thermodynamic effect, leads to a reduction in the saturation pressure associated with the reduced temperature of the liquid, thereby further impeding the phase transition process. Due to the significant differences in thermal properties, the thermodynamic effects in thermosensitive fluids such as high-temperature water and cryogens are more pronounced compared to those in ambient-temperature water. [30] Several researchers have undertaken studies in this area. Li *et al.* developed a cavitation model suitable for organic fluids, incorporating thermodynamic effects, to characterize essential cavitation parameters, including bubble growth rates and bubble number densities. They conducted simulation analyses of transient cavitating flows and corroborated computation precision of the model through comparative validation with experimental data. [31] Yang *et al.* focused on spherical cavitation bubbles, employing the pseudopotential and thermal multi-relaxation-time lattice Boltzmann method to capture and analyze the temperature evolution associated with cavity rupture. They also discussed the temperature, velocity, and pressure distributions within the cavitating flow domain influenced by thermodynamic factors. [32] To study the inhibitory effects of thermal effects under low Reynolds number flow conditions, Le *et al.* conducted simulation studies on the laminar vortex cavitating flow behavior in high-temperature water, further analyzing the impact of these inhibitory effects on flow mechanisms, generated loads, and vortex shedding frequency. [33] Ge *et al.* conducted a series of experimental researches on hydraulic cavitation dynamics in high-temperature water, investigating the combined effects of Reynolds effect and thermodynamic effect on cavitation characteristics. Through visualization experiments capturing cavitating flow, it was discovered that with the increase in temperature, the length of the cavitation cloud first increases and then decreases. By analyzing the cavitation shedding frequency and intensity, they identified the optimal parameter conditions for enhancing cavitation treatment intensity. [34], [35] Additionally, researchers have also investigated the cavitation characteristics of cryogens. Liang *et al.* applied modal decomposition analysis to study the bubble dynamics evolution in liquid nitrogen cavitating flow, exploring the effects of heat transfer on cavity structure and collapse processes. Their results are largely consistent with those of Ge *et al.*, indicating that thermal effects significantly alter the shedding behavior of cavitation clouds. [36], [37] Zhu *et al.* conducted a series of investigations on the cavitation of cryogenic fluids, proposing a numerical method that considers the compressibility and thermodynamic effect of the vapor. They investigated the cavitation behaviors of cryogenic fluids like liquid hydrogen and liquid nitrogen, analyzing the influence of reentrant jets and vortices on cavitating flow. [38], [39] Wei *et al.* conducted numerical studies on the unsteady cavitating flows of liquid nitrogen in a VT under different thermal cavitation modes. The condensation shock wave propagation was observed to be triggered by the impingement of collapse-induced pressure waves from the previously shedding cloud. Furthermore, the interaction between cavitation, vortex dynamics, and entropy production rates was explored in detail. [40].

A review of the literature indicates that while there has been considerable success in academic research pertaining to numerical simulations, most studies focusing on cavitating flow patterns and cavity shedding mechanisms primarily target common temperature working fluids like water and cryogenic fluids such as liquid nitrogen. However, research on the cavitation instability and thermodynamic effects of refrigerants remains limited. This study considers the impact of thermodynamic effects of R134a refrigerant, and based on an experimentally verified cavitation model, analyzes the unsteady cavitation characteristics under three different cavitation states: fusion cavitation (σ=1.522), supercavitation (σ=1.807), and inception cavitation (σ=2.274).

## 2 Numerical model of R134a refrigerant cavitating flow

### 2.1 Governing equations

This study utilized the mixture model to simulate cavitating flow in a VT. The model treats the two-phase flow as a mixture with the same temperature and momentum, simplifying the continuity and momentum equations of the two phases into a single set of equation for the mixture, which is then solved to obtain the cavitating flow region. The mass continuity equation is as follow:

(1) $$\frac{\partial }{\partial t}\left(\rho_{m}\right)+\frac{\partial }{\partial x_{i}}\left(\rho_{m}u_{i}\right)=0$$

where ρ represents density, with the subscripts *v*, *l* and *m* indicating vapor, liquid and mixture, respectively. $\rho_{m}=\alpha \rho_{v}+\left(1-\alpha \right)\rho_{l}$ is the density of the mixture, *u* is the velocity in the *i*-direction, and α is the vapor volume fraction. The momentum equation for the Mixture model is as follow:

(2) $$\frac{\partial }{\partial t}\left(\rho_{m}u_{i}\right)+\frac{\partial }{\partial x_{j}}\left(\rho_{m}u_{i}u_{j}\right)=-\frac{\partial p}{\partial x_{i}}+\frac{\partial }{\partial x_{j}}\mu_{m}\left[\left(\frac{\partial u_{i}}{\partial x_{j}}+\frac{\partial u_{j}}{\partial x_{i}}\right)-\frac{2}{3}\delta_{\mathrm{ij}}\frac{\partial u_{k}}{\partial x_{k}}\right]+f_{i}$$

Here, μ is the viscosity, with the mixture viscosity defined as $\mu_{m}=\alpha \mu_{v}+\left(1-\alpha \right)\mu_{l}$, *p* is the pressure, and *f_i_* represents body forces. $\delta_{\mathrm{ij}}$ is the Kronecker delta, being 0 when i=j and 1 when i≠j. The energy equation for Mixture model is:

(3) $$\frac{\partial }{\partial t}\left(\rho_{m}c_{p,m}T\right)+\frac{\partial }{\partial x_{j}}\left(\rho_{m}c_{p,m}u_{j}T\right)=\frac{\partial }{\partial x_{j}}\left[\left(\lambda_{m}+\lambda_{t}\right)\frac{\partial T}{\partial x_{j}}\right]+S_{E}$$

where λ represents thermal conductivity, with $\lambda_{m}$ and $\lambda_{t}$ indicating the thermal conductivity of mixture and turbulence, respectively, and $c_{p,m}$ is the latent heat of the mixture.

### 2.2 Turbulence model

LES involves filtering the Navier-Stokes equations within a localized spatial domain, separating turbulence into large-scale laminar fluctuations and small-scale turbulent fluctuations. This process is achieved using a low-pass filter. After filtering, the momentum control equation Eq. (2) can be rewritten as follow:

(4) $$\frac{\partial {\overset{¯}{u}}_{i}}{\partial t}+\frac{\partial {\overset{¯}{u}}_{i}{\overset{¯}{u}}_{j}}{\partial x_{j}}=-\frac{\partial \overset{¯}{p}}{\partial {\overset{¯}{x}}_{i}}+v\frac{\partial^{2}{\overset{¯}{u}}_{i}}{\partial x_{i}\partial x_{j}}+\frac{\partial \left({\overset{¯}{u}}_{i}{\overset{¯}{u}}_{j}-\overset{¯}{u_{i}u_{j}}\right)}{\partial x_{j}}$$

where $\overset{¯}{u_{i}}$ is the filtered velocity, and $\overset{¯}{p}$ is the filtered pressure. The subcell scale (SGS) model is introduced:

(5) $$\overset{¯}{\tau_{\mathrm{ij}}}=\left(\overset{¯}{u_{i}}\overset{¯}{u_{j}}-\overset{¯}{u_{i}u_{j}}\right)$$

Here, $\overset{¯}{\tau_{\mathrm{ij}}}$ is defined as SGS stresses. It is assumed that SGS stresses are proportional to the rate-of-strain tensor for the resolved scale $\overset{¯}{S_{\mathrm{ij}}}$, establishing the influence of small-scale vortex on large-scale vortex:

(6) $$\overset{¯}{\tau_{\mathrm{ij}}}=\frac{1}{3}\tau_{\mathrm{kk}}\delta_{\mathrm{ij}}-2\mu_{t}\overset{¯}{S_{\mathrm{ij}}}$$

(7) $$\overset{¯}{S_{\mathrm{ij}}}=\frac{1}{2}\left(\frac{\partial {\overset{¯}{u}}_{i}}{\partial x_{j}}+\frac{\partial {\overset{¯}{u}}_{j}}{\partial x_{i}}\right)$$

In this context, $\tau_{\mathrm{kk}}$ is the isotropic part of the SGS stresses and $\mu_{t}$ is the SGS turbulence viscosity. Presently, the Wall-Adapting Local Eddy-Viscosity (WALE) model is applied to predicting turbulent flow.[41] the turbulent eddy viscosity $\mu_{t}$ is expressed as:

(8) $$\mu_{t}=\rho L_{s}^{2}\frac{{\left(S_{\mathrm{ij}}^{d}S_{\mathrm{ij}}^{d}\right)}^{\frac{3}{2}}}{{\left(\overset{¯}{S_{\mathrm{ij}}}\cdot \overset{¯}{S_{\mathrm{ij}}}\right)}^{\frac{5}{2}}+{\left(S_{\mathrm{ij}}^{d}S_{\mathrm{ij}}^{d}\right)}^{\frac{5}{4}}}$$

In this model:

(9) $$S_{\mathrm{ij}}^{d}=\frac{1}{2}\left({\overset{¯}{g}}_{\mathrm{ij}}^{2}+{\overset{¯}{g}}_{\mathrm{ij}}^{2}\right)-\frac{1}{3}\delta_{\mathrm{ij}}{\overset{¯}{g}}_{\mathrm{kk}}^{2}$$

(10) $${\overset{¯}{g}}_{\mathrm{ij}}=\frac{\partial {\overset{¯}{u}}_{i}}{\partial x_{j}}$$

(11) $$L_{S}=\min\left(\kappa d,C_{W}\Delta \right)$$

where $L_{S}$ is the mixing length for SGS, *κ* is the von Kármán constant, *d* is the distance to the closest wall, $C_{W}$ is the WALE constant with the value of 0.325 in this numerical case, and Δ is the local cell size. Compared to other models, the WALE model better predicts the transition from laminar to turbulent flow and reduces the introduction of excess vorticity in laminar region

### 2.3 Cavitation model

In the study of cavitation in R134a refrigerant, the vapor exhibits significant compressibility, while the liquid refrigerant demonstrates thermal expansion characteristics, with its density varying notably with temperature. To describe the cavitating flow of R134a refrigerant, a cavitation model based on the transport equation model is selected. The transport equation for unit volume fraction can be described as:

(12) $$\frac{\partial }{\partial t}\left(\rho_{v}\alpha_{v}\right)+\frac{\partial }{\partial x_{j}}\left(\rho_{v}\alpha_{v}u_{j}\right)={\dot{m}}^{+}-{\dot{m}}^{-}$$

where ${\dot{m}}^{+}$ and ${\dot{m}}^{-}$ represent the mass of evaporation and condensation per unit time, respectively. Ignoring the second-order terms in the Rayleigh-Plesset equation, surface tension, liquid viscosity, and the effects of non-condensable gases, [42] the relationship between bubble radius and pressure is:

(13) $$\frac{\mathrm{dR}}{\mathrm{dt}}=\pm \sqrt{\frac{2}{3}\frac{\left|p_{\mathrm{sat}}-p\right|}{\rho_{l}}}$$

Here, the mass transfer due to phase change is expressed as:

(14) $$\dot{m}=\frac{\rho_{v}\rho_{l}}{\rho_{m}}\frac{d}{\mathrm{dt}}\left(\frac{4}{3}\pi R^{3}\right)$$

Schnerr and Sauer proposed a cavitation model based on the mass transport equation. [43] They posited that the vapor volume fraction, denoted as *α_v_*, can be represented by the bubbles volume.

(15) $$\alpha_{v}=\frac{\frac{4}{3}\pi n_{b}R_{b}^{3}}{1+\frac{4}{3}\pi n_{b}R_{b}^{3}}$$

In this formula, $n_{b}$ is the number of bubbles per unit volume. The bubble radius $R_{b}$ can be described as:

(16) $$R_{b}=\sqrt[{3}]{\frac{3\alpha_{v}}{4\pi n_{b}\left(1-\alpha_{v}\right)}}$$

Finally, the mass transfer rate can be defined as:

(17) $$\left\{\begin{array}{c} {\dot{m}}^{+}=F_{\mathrm{evap}}\frac{\rho_{v}\rho_{l}}{\rho_{m}}\frac{3\alpha_{v}\left(1-\alpha_{v}\right)}{R_{b}}\sqrt{\frac{2}{3}\frac{\left(p_{\mathrm{sat}}-p\right)}{\rho_{l}}}\left(p<p_{\mathrm{sat}}\right)\\ {\dot{m}}^{-}=F_{\mathrm{cond}}\frac{\rho_{v}\rho_{l}}{\rho_{m}}\frac{3\alpha_{v}\left(1-\alpha_{v}\right)}{R_{b}}\sqrt{\frac{2}{3}\frac{\left(p-p_{\mathrm{sat}}\right)}{\rho_{l}}}\left(p\geq p_{\mathrm{sat}}\right) \end{array}\right)$$

In this equation, $F_{\mathrm{evap}}$ and $F_{\mathrm{cond}}$ are empirical coefficients for the evaporation and condensation processes, typically taken as 1 and 0.2, respectively. The mass transfer rate is proportional to $\alpha_{v}\left(1-\alpha_{v}\right)$ and approaches zero when $\alpha_{v}=0$ and $\alpha_{v}=1$. [44].

### 2.4 Numerical setup

**Fig. 1:**
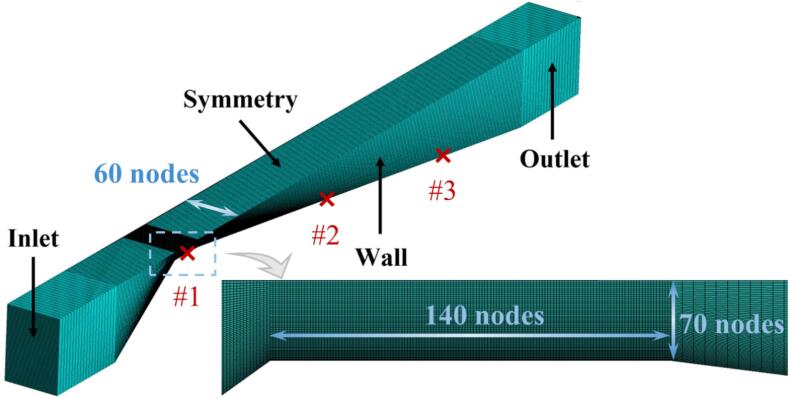
The computational domain details.

Considering the computational complexity and the geometric symmetry in the z-direction, only half of the VT was modeled. The computational domain of the VT, as shown in Fig. 1, is 321 mm long, 10 mm wide, with an inlet height of 16 mm and a throat height of 1.6 mm. The two-dimensional computational domain represents the mid-section of the three-dimensional domain. The structured quadrilateral mesh of the two-dimensional domain comprises over 2.9 × 10^5^ elements, while the structured hexahedral mesh in the three-dimensional domain exceeds 1.657 × 10^7^ elements. The mesh near the walls is refined to mitigate the influence of the walls, ensuring *y+*≤1.

In this study, ANSYS Fluent was used to perform numerical simulations of the steady and unsteady cavitating flow of R134a refrigerant within a VT. To determine the bubble number density suitable for the R134a refrigerant cavitation, this study employs the Realizable *k-ε* turbulence model to simulate the quasi-steady characteristics of R134a refrigerant cavitation in the two-dimensional domain (such as temperature and pressure distribution during cavitation). However, cavitating flow is unsteady and only exhibits quasi-periodic steady behavior under certain conditions. Steady-state simulations fail to capture the complex internal mechanisms of cavitating flow. Additionally, the *k-ε* model typically over-predicts turbulence viscosity, inhibiting the development of eddies. [45] Therefore, in studying the behaviors of cavitating flow, a combination of the LES turbulence model and the S-S cavitation model is used to simulate unsteady cavitation. The process began with a steady-state solution for single-phase flow (liquid R134a refrigerant), which was then used as the initial condition for solving the transient two-phase cavitating flow. The residuals were set to converge to a value of 10^−5^.

A second-order implicit strategy was employed for temporal discretization, while a coupled algorithm was used for velocity–pressure coupling. The second-order upwind scheme was applied to discretize the energy equations, and the momentum equation was discretized using the bounded central differencing scheme. For the spatial discretization of pressure, density, and volume fraction, the PRESTO! scheme, first-order upwind strategy, and QUICK schemes were utilized, respectively.

The time step was set to 5 × 10^−5^s to ensure the flow Courant number remained below 1. A no-slip wall boundary condition was applied to the surface of the flow channel. The inlet and outlet boundary conditions were set as velocity-inlet and pressure-outlet, respectively, based on the experimental operating conditions. The conditions involved in this study are outlined in Table 1.

**Table 1:** Flow conditions for numerical and experimental sets.

Sets	*σ*	Flow velocity	Upstream temperature and pressure	Saturation pressure	Reynolds number
1	1.522	2.55 m/s	288.3 K/ 788 kPa	491 kPa	4.59 × 10^5^
2	1.807	1.76 m/s	288.3 K/ 788 kPa	491 kPa	3.17 × 10^5^
3	2.274	1.59 m/s	288.3 K/ 788 kPa	491 kPa	2.86 × 10^5^
4	2.274	1.59 m/s	277.4 K/ 584 kPa	341 kPa	3.30 × 10^4^
5	1.522	2.55 m/s	277.4 K/ 584 kPa	341 kPa	5.29 × 10^4^

In the simulations, liquid R134a refrigerant and vapor R134a refrigerant were defined as the primary and secondary phases, respectively. Different from water cavitation, the cavitation process of R134a is typically accompanied by larger temperature changes. Therefore, during the simulation process, it is essential to consider the changing physical properties of R134a refrigerant, as detailed in Table 2. The thermophysical properties of R134a refrigerant are approximated as polynomial functions of temperature:

(18) $$f\left(T\right)=A_{0}+A_{1}T+A_{2}T^{2}+A_{3}T^{3}+A_{4}T^{4}+A_{5}T^{5}\left(240K<T<323K\right)$$

**Table 2:** Coefficients of thermophysical properties.

Parameters	*A_0_*	*A_1_*	*A_2_*	*A_3_*	*A_4_*	*A_5_*
$\rho_{l}/kg\cdot m^{-3}$	4.0003e3	−4.2631e1	3.259e-1	−1.328e-3	2.717e-6	−2.265e-9
$\rho_{v}/kg\cdot m^{-3}$	−1.8410e3	3.6836e1	−2.951e-1	1.188e-3	−2.418e-6	2.019e-9
$Cp_{l}/J\cdot kg^{-1}\cdot K^{-1}$	−1.5127e4	3.2760e2	−2.6440	1.069e-2	−2.163e-5	1.7582e-8
$Cp_{v}/J\cdot kg^{-1}\cdot K^{-1}$	−3.1507e4	6.3556e2	−5.0450	2.005e-2	−3.981e-5	3.171e-8
$\lambda_{l}/W\cdot m^{-1}{\cdot K}^{-1}$	3.478e-1	−2.263e-3	1.068e-5	−3.548e-8	6.673e-11	−5.427e-14
$\lambda_{v}/W\cdot m^{-1}{\cdot K}^{-1}$	−2.403e-1	4.697e-3	−3.709e-5	1.491e-7	−2.999e-10	2.421e-13
$\mu_{l}/kg\cdot m^{-1}\cdot s^{-1}$	5.845e-2	9.520e-4	6.353e-6	−2.149e-8	3.665e-11	−2.514e-14
$\mu_{v}/kg\cdot m^{-1}\cdot s^{-1}$	−7.842e-5	1.630e-6	−1.294e-8	5.288e-11	−1.088e-13	8.995e-17
$P_{\mathrm{sat}}/Pa$	3.0429e6	−7.0833e4	6.2891e2	−2.5883	4.5758e-3	−2.0051e-6

## 3 Experimental setup and model validation

### 3.1 Test rig

**Fig. 2:**
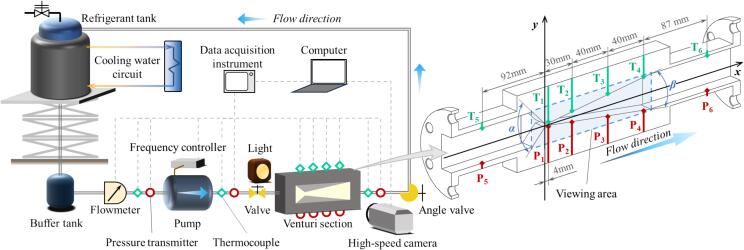
Schematic of test system and tested Venturi.

Due to its simplicity, VT is often used as an ideal component for studying cavitation. As shown in Fig. 2, a visualized closed hydraulic cycle system is constructed for studying the cavitation of the refrigerant in a VT. Additionally, a detailed cross-sectional diagram of the VT is provided on the right side of the Fig. 2. During operation, the working medium in the refrigerant tank is pumped into the VT, where cavitation occurs at its throat. The flow details are observable through the acrylic side walls. A frequency controller connected to the pump allows for flow velocity adjustment, and flow rate can be controlled by the angle valve opening. To prevent unstable operation of the pump, a buffer tank is installed upstream of the pump. The liquid level in the refrigerant tank can be raised with a lifter during experiments to elevate the pressure at the pump inlet, and the temperature of the refrigerant in the loop can be regulated via a cooling water circuit. All pipelines are insulated to ensure the working medium is a saturated liquid.

The VT used in the experiment is made of stainless steel, featuring a throat length of 8 mm, height of 3.2 mm, an inlet convergence angle *α* of 92°, and an outlet diffusion angle *β* of 15°. Six pressure transducers (range 0–1.6 MPa, accuracy 0.2 % F.S.) and six T-type thermocouples (accuracy 0.15 K) are utilized to gauge the static pressure and temperature in the test section, with their positions shown in the right diagram of Fig. 2. The flow rate in the cycle loop is measured using a vortex flowmeter, with a range of 0.417–4.17 L/s and accuracy of 0.5 % F.S. The pump driving the hydraulic cycle, developed by the research team [46], is a high-speed centrifugal pump. At its rated operating condition (speed 7500 rpm, flow rate 0.98 L/s), it can provide a delivery pressure of 4.4 bar. A Phantom Miro R310 high-speed camera is applied for visualization of the test section, with enhanced image contrast using a strong light source. At a recording speed of 13,000 frames per second, the shooting resolution is 640 × 480, with an exposure time of 2 μs. Further details are available in the preceding work for reference. [47].

The cavitation number *σ* is commonly employed to characterize the intensities of cavitating flow. It can be defined as follow:

(19) $$\sigma =\frac{p_{\mathrm{in}}-p_{v}\left(T_{\mathrm{in}}\right)}{p_{\mathrm{in}}-p_{\mathrm{out}}}$$

where $p_{\mathrm{in}}$ is the inlet pressure, and $p_{v}\left(T_{\mathrm{in}}\right)$ is the saturation pressure corresponding to the inlet temperature. Assuming temperature errors affect the viscosity, density, and vapor pressure properties of the working fluid in a simplified first-order linear relationship, the uncertainty of the cavitation number in the experiments can be determined using the Eq. (20). The range is between 0.0185 and 0.0288.

(20) $$\frac{\Delta \sigma }{\sigma }=\sqrt{{\left(\frac{P_{\mathrm{in}}}{P_{\mathrm{in}}-P_{v}\left(T_{\mathrm{in}}\right)}\right)}^{2}{\left(\frac{\Delta P_{\mathrm{in}}}{P_{\mathrm{in}}}\right)}^{2}+{\left(\frac{P_{v}\left(T_{\mathrm{in}}\right)}{P_{\mathrm{in}}-P_{v}\left(T_{\mathrm{in}}\right)}\right)}^{2}{\left(\frac{\Delta T_{\mathrm{in}}}{T_{\mathrm{in}}}\right)}^{2}+{\left(\frac{P_{\mathrm{in}}}{P_{\mathrm{in}}-P_{\mathrm{out}}}\right)}^{2}{\left(\frac{\Delta P_{\mathrm{in}}}{P_{\mathrm{in}}}\right)}^{2}+{\left(\frac{P_{\mathrm{out}}}{P_{\mathrm{in}}-P_{\mathrm{out}}}\right)}^{2}{\left(\frac{\Delta P_{\mathrm{out}}}{P_{\mathrm{out}}}\right)}^{2}}$$

### 3.2 Validation of the cavitation model

In the S-S cavitation model, the bubble number density $n_{b}$​ is typically set to 10^13^, a value that has been extensively validated in cavitation studies using ambient temperature water. [48] For cryogenic fluids like liquid hydrogen and liquid nitrogen, $n_{b}=10^{8}$ has been experimentally verified. [44] Considering the substantial variations in properties like density, viscosity, and surface tension among various working fluids, it is necessary to modify the bubble number density $n_{b}$ in the S-S model to ensure consistency between simulation and experimental results.

**Fig. 3:**
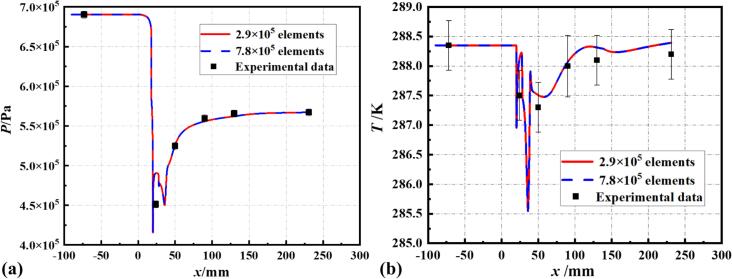
Verification of the computational model based on wall pressure and temperature.

In this study, several sets of conditions with different temperatures and cavitation numbers were chosen for simulation, aligning the experimental test conditions with the simulations. [49] As shown in Fig. 3, Set 1 was used for element independence verification, with the bubble number density $n_{b}$ set at 10^9^. The results indicate that the pressure and temperature distributions with 2.9 × 10^5^ and 7.8 × 10^5^ elements are essentially consistent with the experimental data. To save on computational costs, subsequent simulation studies will be conducted on 2.9 × 10^5^ element.

**Fig. 4:**
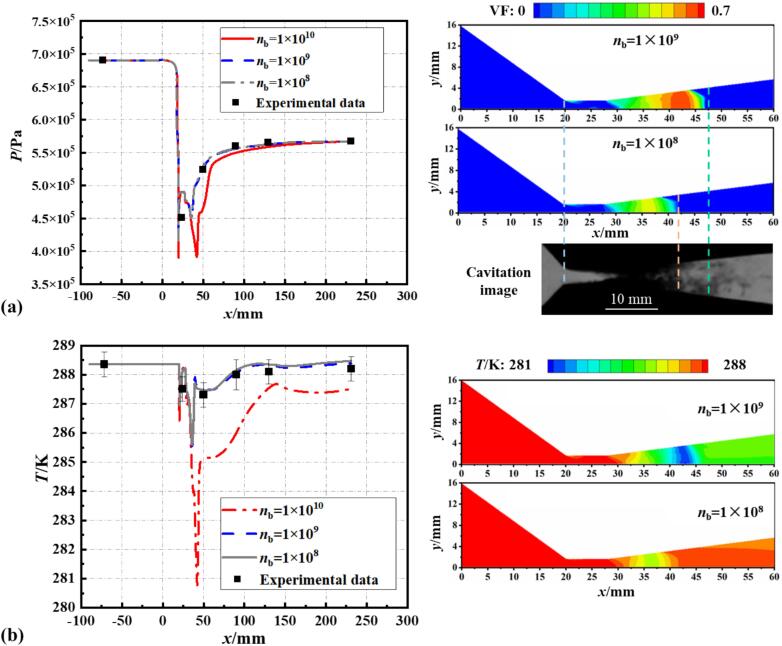
The impact of varying bubble number densities on (a) pressure and (b) temperature of the wall, compared with experimental data.

Fig. 4 illustrates the simulations conducted under Set 1 conditions, using a non-isothermal computational model with the bubble number density $n_{b}$ set to 10^8^, 10^9^, and 10^10^. When $n_{b}$​ exceeds 10^10^, the simulation results become unstable and fail to converge. At $n_{b}=10^{10}$, it is evident from the figure that the pressure and temperature distributions along the wall significantly deviate from the experimental data. However, for $n_{b}=10^{8}$ and $n_{b}=10^{9}$, the pressure and temperature distribution curves align more closely with the experimental values, with the temperature curve in the diffusion section at $n_{b}=10^{9}$ being closer to the experimental values.

Further insights are gained from the vapor volume fraction and temperature distribution. Comparing with the cavitation images captured in experiments, it is noted that at $n_{b}=10^{8}$, the cavitation field length is only 22.3 mm, exhibiting a 29 % discrepancy from the 28.8 mm measured experimentally. However, the cavitation field length is 27.4 mm at $n_{b}=10^{9}$, with an error of 5.1 % relative to the experimental results. Additionally, at $n_{b}=10^{9}$, the degree of cavitation is more severe, and a greater temperature drop is observed within the cavitation cloud.

**Fig. 5:**
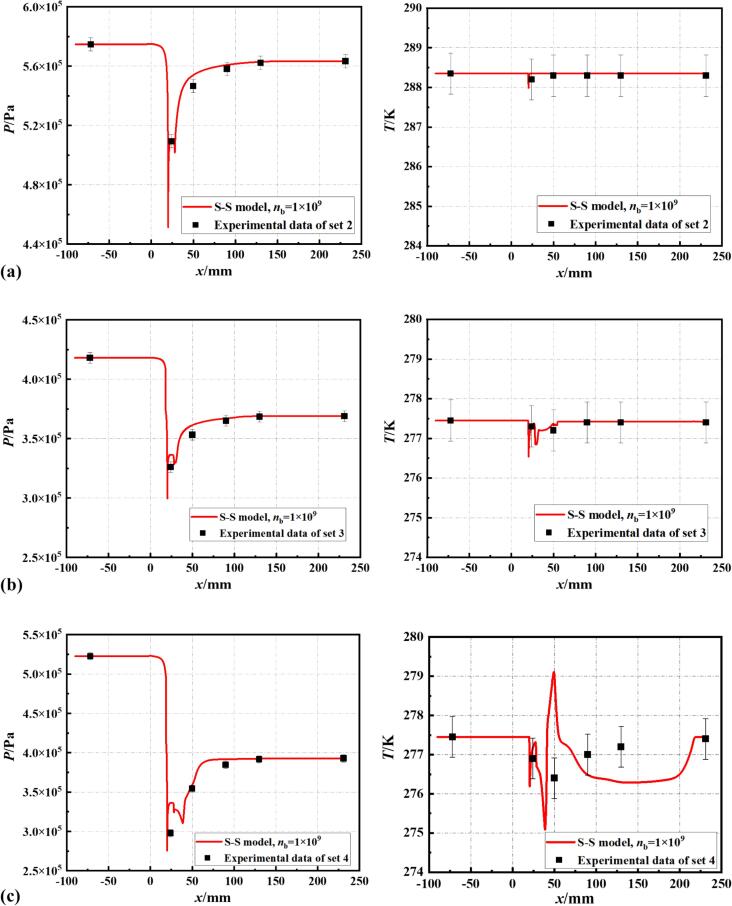
The discrepancy between simulated results and experimental data for pressure and temperature distribution on the wall at (a) Set 2, (b) Set 4 and (c) Set 5 conditions.

Based on the comparative analysis of pressure and temperature distribution curves and cavitation length with experimental data, this study sets the bubble number density in the S-S cavitation model at 10^9^. The non-isothermal simulation model, with this improved bubble number density, predicts pressure and temperature within the VT that are in good agreement with experimental data. As illustrated in Fig. 5, this modified cavitation model with the revised bubble number density remains effective in accurately predicting cavitating flow under different cavitation conditions within the temperature range of 278 K to 288 K (Set 2, Set 4, and Set 5).

## 4 Results and discussions

### 4.1 Unsteady behavior of cavitating flow

Under varying conditions, the shedding modes of cavitating flow within the VT exhibit distinct characteristics. A comparison between experimental observations of cavitating flow and numerical simulations was conducted, utilizing direct imagery captured by the high-speed camera and visualized simulation results. The visualization of cavitation simulations aligns closely with the experimental evolution of cavities, as shown in Table 3. The extracted three-dimensional models, represented by 50 % isosurfaces of vapor volume fraction, closely approximate the results calculated from two-dimensional models. To facilitate comparison and illustrate flow mechanisms while conserving computational resources, a portion of the research results is conducted by analyzing results from two-dimensional models.

**Table 3:** Comparison of cavitating flow captured in both transient simulation and experimental images.

**Fig. 6:**
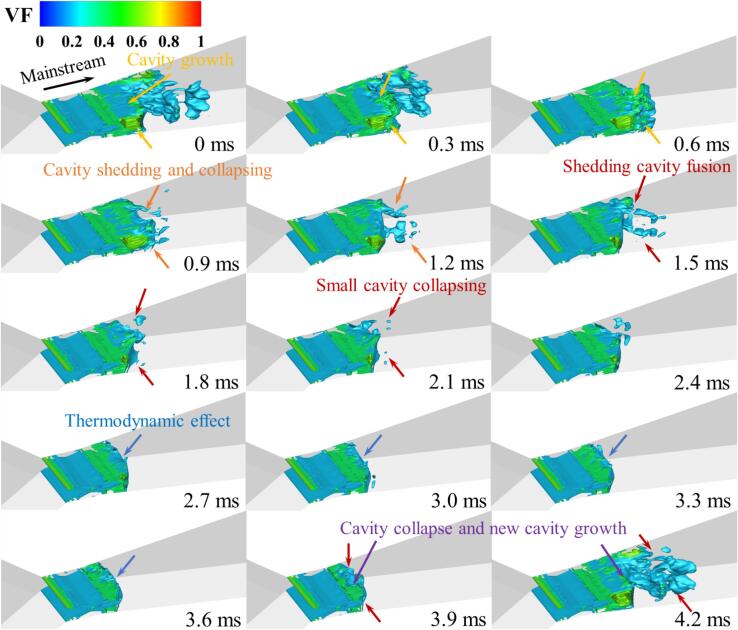
Spatiotemporal evolution of cavities in fusion cavitating flow (Set 1 condition).

**Fig. 7:**
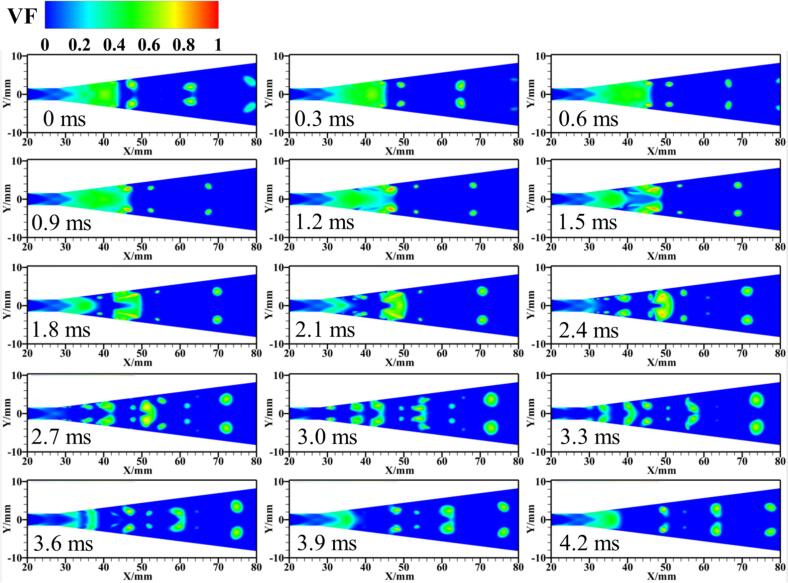
The temporal evolution of cavities in the 2D model under Set 1 conditions.

Further analysis of the transient flow process of R134a is conducted, with Fig. 6 and Fig. 7 presenting 15 snapshots of the instantaneous vapor volume distribution under Set 1 conditions at a time interval of 0.3 ms for 3D and 2D calculations, respectively. It is observed that in most cavitation regions, the vapor volume fraction is less than 0.7, with values around 0.8 occurring only at the core of the shedding cloud clusters. This phenomenon is consistently observed in both 3D and 2D computations.

These cavitation clouds exhibit a fusion cavitation behavior. From 0 ms to 0.9 ms, the main cavitation area maintains a stable blunt body shape (see Fig. 6), with only a few small bubble clouds detaching from the rear of the cavitation zone and gradually collapsing.

In contrast to the previous phase, from 1.2 ms to 3.0 ms, the cavitation clouds attached to the walls of the Venturi tube detach symmetrically from both sides at the leading edge of the blunt body in the mainstream and gradually merge downstream, forming sheet cavitation clouds (see Fig. 7). During this period, the blunt body in the mainstream is affected by the high-pressure wake of the detaching sheet cavitation clouds, resulting in partial collapse at the leading edge and further deformation of the blunt body shape.

Between 3.3 ms and 4.2 ms, as the detaching sheet cavitation clouds move downstream and collapse, newly formed cavitation clouds gradually merge, developing into new blunt body-like cavitation clouds in the main cavitation area. A comparison of the transient results from the 3D and 2D calculations further validates the consistency between the two models under a similar setup.

**Fig. 8:**
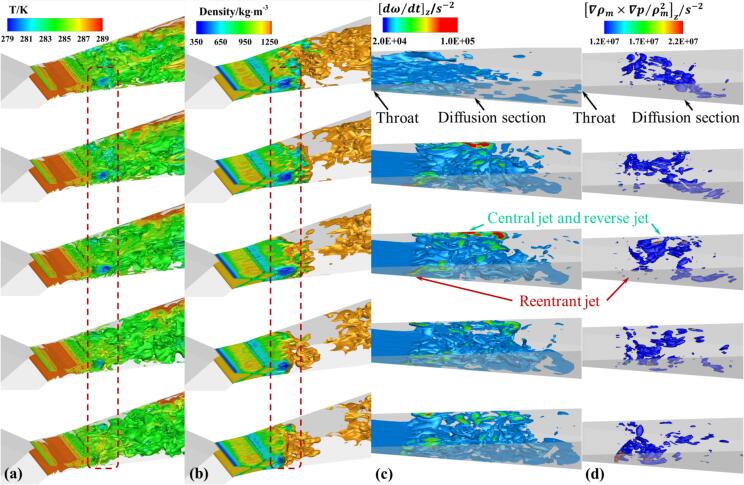
The distribution of (a) temperature, (b) density, (c) vorticity and (d) baroclinic torque term in Set 1 cavitation region.

Under Set 1 conditions, the distribution of temperature, density, and the vorticity transport value $D\vec{\omega }/Dt$​ in the cavitation area is displayed in Fig. 8. Due to the pressure in the cavitation core region being lower than the saturation pressure corresponding to the local temperature, it can be observed from Fig. 6 and Fig. 8a that the temperature in the core region is also significantly low (as indicated by the red box), with a temperature difference of up to 7 K compared to the upstream fluid. As cavitation progresses, the core region absorbs heat from the surrounding fluid, causing a drop in the temperature of the surrounding flow. Simultaneously, the corresponding saturation vapor pressure in the cavitation region decreases, thereby suppressing the cavitation process of the refrigerant. In areas of severe cavitation, the higher the vapor fraction of the refrigerant, the lower its density, as depicted in Fig. 8b. For most detached cavitation clouds, the internal density gradient is low, but the density changes dramatically at the edges of detachment. In conjunction with Fig. 8c, it is evident that there are certain vortices at the center of the cavitation clouds, maintaining lower pressure within the cloud center. This promotes the phase change process in the surrounding liquid, leading to decreased temperature and density. As the cloud enters the collapse area, due to the increased ambient pressure, the shedding cloud begins to collapse and condense. The heat released during condensation paradoxically causes the temperature of the liquid surrounding these cavitation clouds to rise, and the local liquid temperature might exceed the inflow temperature. In the conditions discussed in this research, the maximum temperature increase is up to 0.6 K, compared to the 1.4 K increase recorded in the Ref. [50].

During the development of the cavitating flow, small cavities continuously form and detach from the tail of the main cavitation area or near the walls. These formations and collapses are predominantly vortex-driven, hence the introduction of the vorticity transport equation:

(21) $$\frac{D\vec{\omega }}{\mathrm{Dt}}=\left(\vec{\omega }\cdot \nabla \right)\vec{V}-\vec{\omega }\left(\nabla \cdot \vec{V}\right)+\frac{\nabla \rho_{m}\times \nabla p}{\rho_{m}^{2}}+\left(v_{m}+v_{t}\right)\nabla^{2}\vec{\omega }$$

In this equation, $D\vec{\omega }/Dt$ represents the total derivative of vorticity with respect to time, reflecting the rate of vorticity transport, as shown in Fig. 8c. The term $\left(\vec{\omega }\cdot \nabla \right)\vec{V}$ signifies the stretching and deformation of vortex structures caused by velocity gradients, while $\vec{\omega }\left(\nabla \vec{\cdot V}\right)$ represents the impact of fluid parcel expansion and contraction on vorticity. The term $\nabla \rho_{m}\times \nabla p/\rho_{m}$ is the baroclinic torque, characterizing vorticity changes caused by misalignment of pressure and velocity gradients, as seen in Fig. 8d. The final term, $\left(\nu_{m}+\nu_{t}\right)\nabla^{2}\vec{\omega }$, is the viscous dissipation term, representing vorticity changes due to viscous dissipation.

As the gas–liquid mixed R134a refrigerant moves from the throat to the diffusion section of the VT, two vortex driven cavity shedding modes are observed. One mode is initiated by the combined influence of reentrant flow from the wall and the centrifugal effect of vortices, resulting in an increase in vorticity. This occurs near the walls, from the throat to the front part of the diffusion section. Influenced by reentrant flow, there is a significant increase in the absolute value of the baroclinic torque, which in turn elevates the local absolute vorticity, leading to the generation of more vortices. The centrifugal action of these vortices reduces internal pressure, causing more liquid to cavitate around them. Consequently, the vapor fraction in the cavitation area increases, rapidly reducing the fluid density, thus further increasing vorticity. Additionally, the thermodynamic effect of refrigerant, which lowers the temperature of the surrounding liquid, inhibits further development of cavitation. This process is distinctly evident between 2.7 ms and 3.9 ms in Fig. 6, and similar flow mechanisms have been observed in cryogenic fluids as well. [51].

The other mode is driven by the interaction of the central jet in the mainstream and the reverse jet generated by collapsing cavities, primarily occurring in the main cavitation region of the VT. As the cavitation clouds are carried downstream by the mainstream flow, the combined effects of the central jet and pressurization cause these large-scale cavitation clouds to collapse and break apart into smaller cavities. The increased absolute value of the annular baroclinic torque within the large-scale cavitation clouds further amplifies the vorticity. Correspondingly, the annular vortices break down into smaller vortex clusters. These clusters create low-pressure areas that sustain the detached cavities moving downstream until they eventually collapse.

**Fig. 9:**
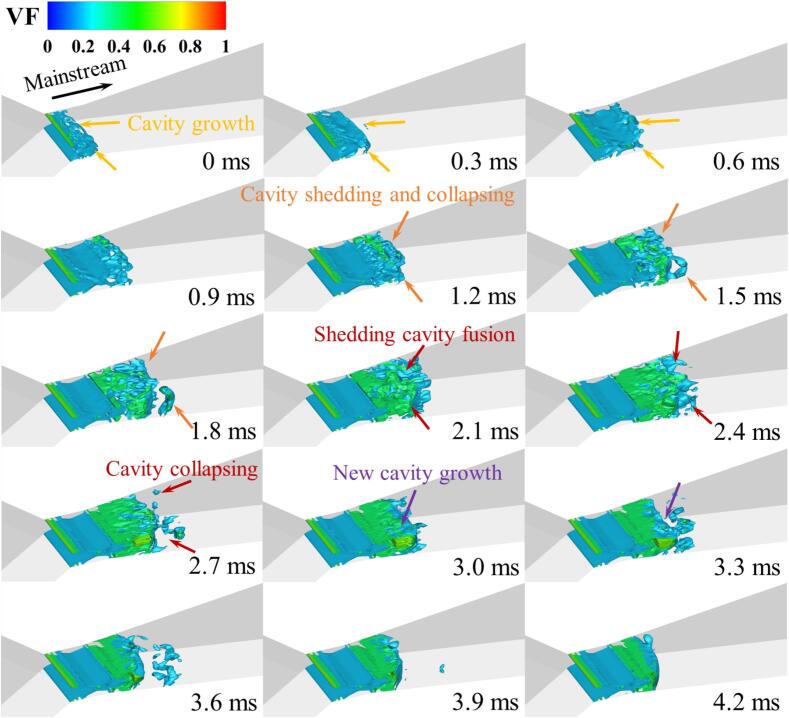
Temporal evolution of cavities in supercavitation flow (Set 2 condition).

Fig. 9 depicts the spatiotemporal changes of the cavitating flow under Set 2 conditions. The cavitation number in this scenario is slightly higher than that in Set 1. The cavitation clouds in the main cavitation area primarily maintain a blunt body shape, with the vapor volume fraction mostly below 0.6, characterizing this as a supercavitation flow. Throughout the formation, development, detachment, and collapse of large-scale cavitation clouds, a notable difference from the previous Set 1condition is observed between 3.0 ms and 3.6 ms. When the cavitation cloud of the next cycle starts to form, the cloud from the previous cycle has already detached and begun collapsing, with minimal interaction between the two cycles.

However, as seen from the cavitation cloud snapshots between 1.2 ms and 3.0 ms in Set 1, when the next cycle cavitation cloud starts to form, the cloud from the previous cycle has not yet fully detached from the main cavitation area. The new cavitation cloud, not fully formed, is swept away by the vortices generated by the cloud from the previous cycle, resulting in smaller-scale detached cloud clusters. Therefore, the cavitation cloud detachment interface in Set 1 is extremely unstable, manifesting non-periodic flow characteristics.

**Fig. 10:**
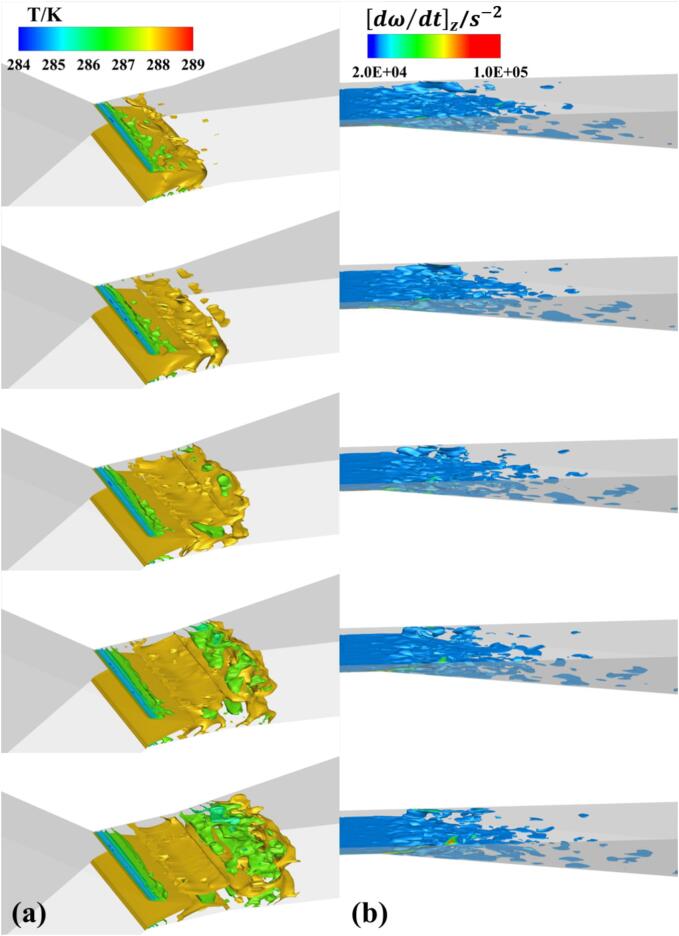
The distribution of (a) temperature and (b) vorticity in Set 2 cavitation region.

Fig. 10 presents the temperature and vorticity changes within the cavitation domain under Set 2 conditions. Clearly, the significant temperature decrease predominantly takes place within the large-scale cavitation clouds, maintained by the low-pressure zones formed by central vortices. As these clouds move downstream, they collapse due to the increasing pressure.

Comparatively, even though Set 1 conditions exhibit more intense cavitation, the cavitation clouds under Set 2 conditions have a more extensive low-temperature region, with the maximum temperature drop reaching 4 K.

**Fig. 11:**
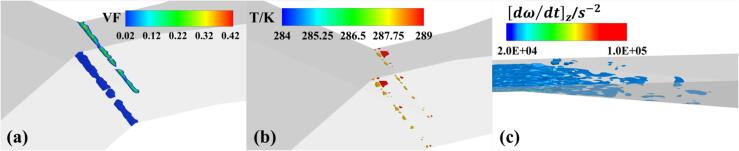
The distribution of (a) bubble volume fraction, (b) temperature and (c) vorticity in Set 3 cavitation region.

Since the cavitation number in Set 3 conditions exceeds the critical threshold, the cavitation region is predominantly composed of small-scale cavitation clouds, characterized by attached cavitation, as shown in Fig. 11. The vapor fraction in these clouds is less than 0.3, with small-scale cavitation clouds continuously forming and collapsing rapidly. The overall cavitation region experiences only a minor temperature drop of less than 1 K, and there is no localized temperature rise at the margin of the cavitation.

Most of the vorticity is confined to the wall area by the mainstream flow. In this condition, the cavitation is primarily generated by the pressure drop in the mainstream, while the vortices sustain the brief presence of small-scale cavitation clouds in the pressure recovery zone. The attached behavior of the cavity indicates a more stable flow pattern compared to the more intense and detached cavitation observed in the other sets.

### 4.2 Impact of thermodynamic effects

The thermodynamic effect varies with different working fluids, determined by the thermophysical properties of the fluid. In cavitation involving ambient temperature water, the fluid temperature remains relatively unchanged, and the process can be assumed isothermal ($p_{\mathrm{sat}}=constant$), with the fluid saturation pressure set as a constant. However, the latent heat and liquid thermal conductivity of R134a refrigerant are much lower (approximately 1/13 and 1/7 of water at 288.3 K, respectively), leading to a more pronounced temperature drop in the phase change area. Additionally, at 288.3 K, the saturation pressure of R134a refrigerant increases by 15,550 Pa/K for every 1 K increase in temperature, compared to water saturation pressure–temperature rate of change of only 107.7 Pa/K. This means that the phase change of R134a refrigerant is more sensitive to temperature drops.

**Fig. 12:**
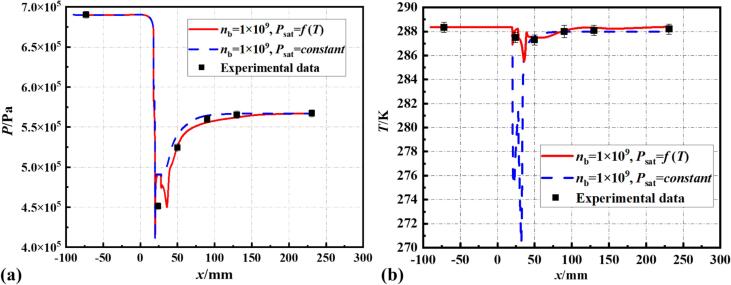
The influence of thermodynamic effect on wall pressure and temperature.

**Fig. 13:**
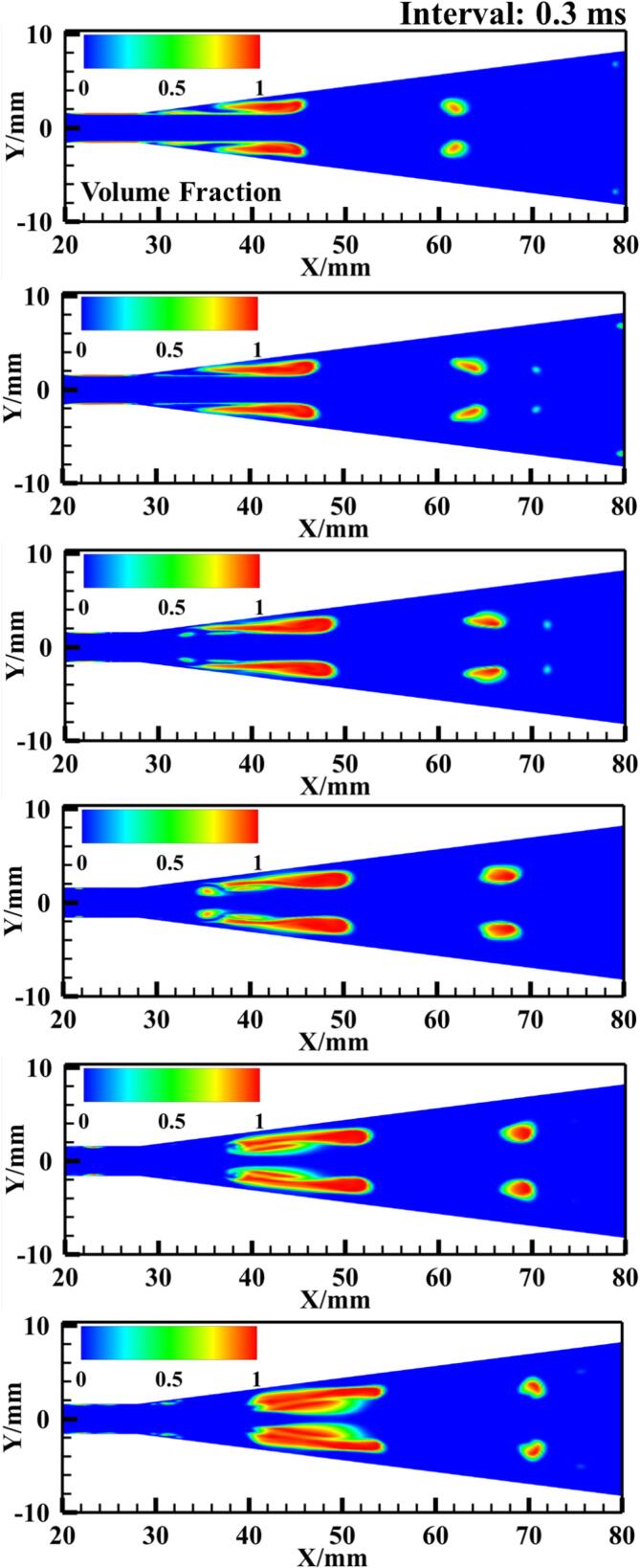
The distribution of vapor volume fraction in the isothermal model.

**Fig. 14:**
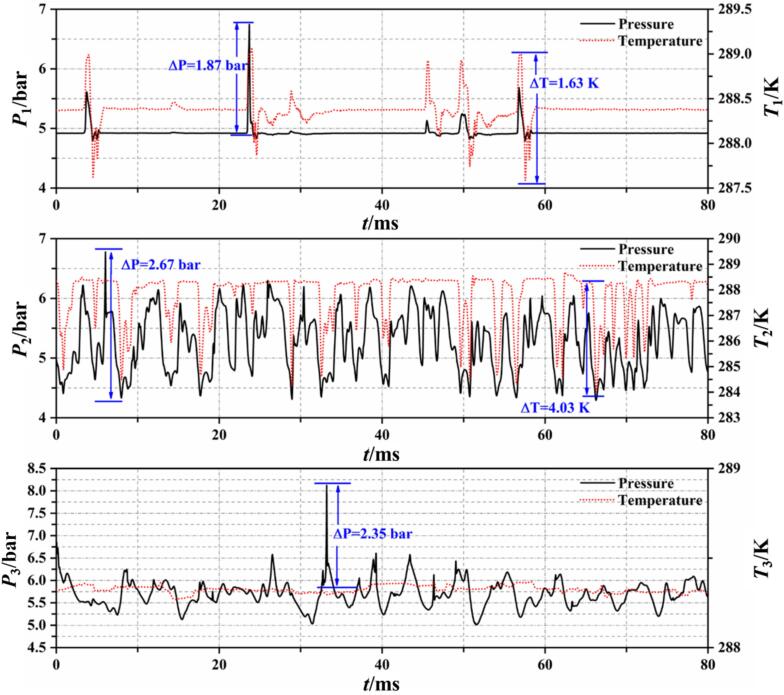
The collapse of cavities leads to fluctuations in wall temperature and pressure fluctuations at Set 1 condition. The #1 monitor is located at the throat of the VT, the #2 monitor at the front of the diffusion section, and the #3 monitor at the rear of the diffusion section.

**Fig. 15:**
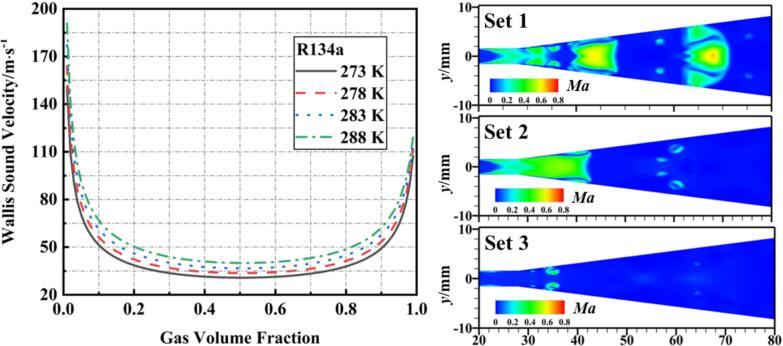
Wallis sound velocity and *Ma* number under various working conditions.

Simulations of Set 1 conditions were conducted using both isothermal and non-isothermal assumptions ($p_{\mathrm{sat}}=f\left(T\right)$), with results shown in Fig. 12, Fig. 13, Fig. 14, Fig. 15. Regardless of the assumption, the pressure distribution along the walls of the VT aligns well with experimental data (see Fig. 12 (a)). However, the wall temperature curves (see Fig. 12 (b)) indicate that the maximum temperature drop reaches 12 K under the isothermal assumption, which significantly deviates from the experimental data. In contrast, the maximum temperature drop is 2.3 K under the non-isothermal assumption, compared to the experimental maximum drop of 1.2 K. The non-isothermal model accounts for the impact of temperature drop on saturation pressure, preventing an over-prediction of the temperature drop during cavitation. In the diffusion section of the VT, the gradual collapse of cavities causes local condensation, slightly increasing the temperature in the collapse area. This aligns with the findings reported in Ref [50] for high-temperature water cavitation experiments.

Analyzing the transient simulation results, with each vapor volume fraction distribution image spaced at 0.3 ms intervals, offers further insights. For the R134a refrigerant vapor volume fraction distribution, the isothermal assumption (see Fig. 13) shows bubbles mainly developing along the walls of the VT, forming a cavitation region with a maximum bubble volume fraction of 0.9 at the walls of the diffusion section. This is consistent with the distinct phase interface between gas and liquid as described in Ref. [52]. However, in the non-isothermal model (see Fig. 7), thermodynamic effects significantly inhibit the development of cavitation clouds, with only a few cavities exceeding a vapor volume fraction of 0.8 at the vortex core of the blunt body of the cavitation clouds. A large amount of gas–liquid mixture fills the throat channel of the VT, forming large cavitation cloud clusters, similar to the mist-like cavitation area caused by thermodynamic effects as proposed in the experimental results of Ref. [53].

Moreover, the two models exhibit different cavitation cloud shedding modes. Under the isothermal assumption, the detachment of large-scale cavitation clouds located at the exit of the VT throat is solely influenced by the reverse jet. The detached cavities flow downstream with the mainstream and collapse, forming a relatively stable cavitation cloud development cycle with a period of 2.8 ms. However, the non-isothermal model simulation reveals a more complex cavitation cloud shedding pattern, with both cavitation cloud detachment from the walls of the VT and partial detachment of the blunt body at the rear, induced by the wall reverse jet. These shedding patterns lead to instability in the cavitation process. Due to the collapse of the partially detached cavities at the blunt body, the cavitation boundary position also continuously changes with the cavitating flow.

Table 4 demonstrates the temperature and vortex distributions for the two assumption models. In the isothermal assumption, the temperature difference between the core of the cavitation cloud and the mainstream liquid reaches up to 50 K, significantly overestimating the temperature drop during cavitation in the entire flow process. Conversely, with the non-isothermal assumption, the maximum reduction in temperature is only 2.5 K.

**Table 4:** The influence of thermal effect on temperature and vorticity distribution.

The vorticity distribution indicates that, under the isothermal assumption, the maximum vorticity occurs inside the cavitation clouds. These vortices are crucial in maintaining the integrity of the large-scale clouds, preventing local detachment. However, in the non-isothermal assumption, due to thermodynamic effects leading to local temperature drops and a decrease in saturation pressure, the blunt body cavitation clouds cannot maintain their shape under the influence of the wall reverse jet. This results in the clouds beginning to detach from the center, leading to high vorticity values at the gas–liquid interface.

### 4.3 Temporal variation characteristic of temperature and pressure

To investigate the energy conversion characteristics of the phase change process of R134a refrigerant, monitoring points were set up on the walls of the numerical model to record temperature and pressure changes during cavitation. The locations of these monitoring points correspond to #1, #2, and #3 in Fig. 1, which are situated at the throat, the front part of the diffusion section, and the rear part of the diffusion section of the VT, respectively.

Fig. 14 shows the temporal variations of temperature and pressure at these monitors, representing distinct cavitation characteristics. In Set 1 conditions, the VT experiences a high degree of cavitation, with continuous generation of cavitation clouds at the throat. The pressure at #1 remains stable at around 4.88 bar, corresponding to the saturation vapor pressure at a temperature of 288 K, indicating a gas–liquid two-phase state. The collapse of a small number of bubbles near this point results in a pressure rise up to 1.87 bar, typically lasting no more than 2 ms. Concurrently, the heat released from bubble collapse causes a temporary increase in the surrounding liquid temperature. This released heat induces further cavitation of the surrounding saturated R134a refrigerant, leading to a decrease in temperature and pressure. After a period of oscillations, the pressure and temperature return to the saturated state. The pressure and temperature at #2 oscillate with the flow of cavitation clouds, decreasing with cavitation occurrence. When cavitation clouds collapse near point #3, the pressure instantaneously rises by 2.35 bar. However, since #3 is located at the rear part of the diffusion section, where fewer collapsing bubbles occur, the pressure changes have minimal impact on the temperature.

Based on the monitoring of wall pressure and temperature, it is observed that changes in pressure and temperature within the cavitation region are synchronous. When cavitation clouds collapse, the pressure rises rapidly, but the corresponding increase in temperature exhibits a lag of 0.1 ms to 0.3 ms. This phenomenon predominantly occurs in the cavitation cloud detachment area under low cavitation number conditions.

Wallis [54] proposed the concept of isenthalpic sound speed for phase change processes based on the critical conditions for flow phase equilibrium:

(22) $$\frac{1}{c_{w}^{2}}=\rho_{m}\left(\frac{\alpha_{v}}{\rho_{v}+c_{v}^{2}}+\frac{\alpha_{l}}{\rho_{l}c_{l}^{2}}\right)$$

Here, $c_{l}$ and $c_{v}$ represent the sound speeds in the liquid and vapor, respectively, while $c_{w}$ is the sound speed in the two-phase flow. Consequently, the ratio of the fluid velocity within the VT to the two-phase sound speed is defined as the local Mach number:

(23) $$\mathrm{Ma}=\frac{v}{c_{w}}$$

Fig. 15 illustrates the variations in *Ma* and two-phase sound speed as a function of the bubble volume fraction under different cavitation conditions. When the vapor volume fraction decreases to 0.2, the corresponding local sound speed is approximately 50 m/s, with a *Ma* around 0.15, and the local fluid velocity is about 18 m/s. Due to the presence of reverse jets and vortices, cavitation predominantly occurs near the walls, where the temperature drop and pressure drop are approximately synchronized.

However, during collapse, as cavitation clouds mostly form away from the walls and the bubble volume fraction swiftly diminishes at the edges of collapsing clouds, the speed of pressure wave propagation (local sound speed) increases swiftly. At this point, since the pressure wave propagation speed is greater than the flow speed (convective heat transfer rate), which in turn is greater than the temperature propagation speed (heat conduction transfer rate), a time-lag of temperature variation occurs.

**Fig. 16:**
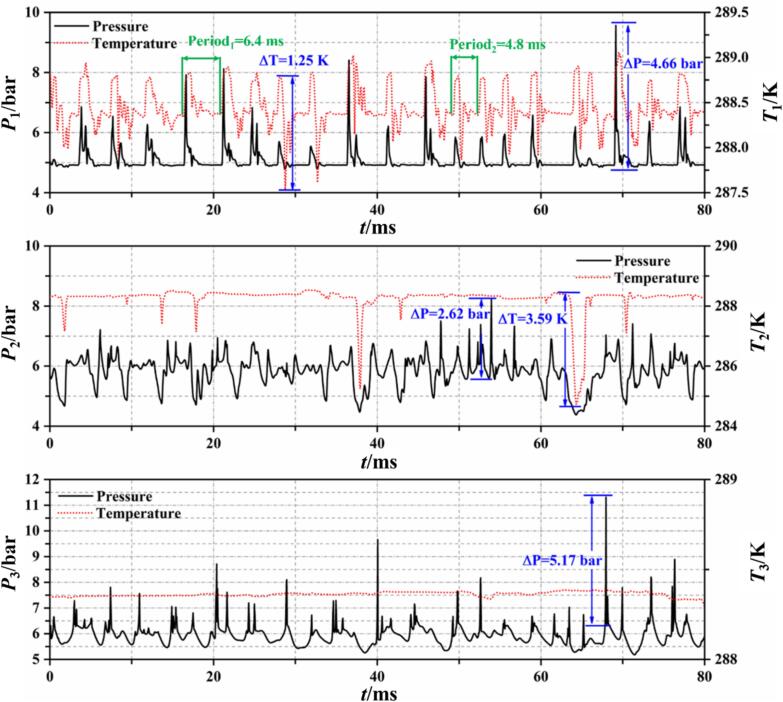
Wall temperature and pressure fluctuations at Set 2 condition.

Fig. 16 shows the temperature–pressure curves at the monitoring points under Set 2 conditions. Due to the periodic detachment and collapse of small-scale cavitation clouds in the throat area, where #1 is located, the temperature and pressure at this point also exhibit periodic spikes followed by oscillations back to the saturation pressure corresponding to the inflow temperature. However, unlike the more distinct periodicity observed in cavitation with water, the detachment-collapse period of R134a refrigerant cavitation is not fixed. Over an 80 ms recording period, each cycle fluctuates between 4.8 ms and 6.4 ms in duration. Similar to Set 1 conditions, both temperature and pressure decrease as cavitation clouds pass #2. Since the degree of cavitation is lower in Set 2, the frequency of temperature and pressure drops is less frequent, with intervals over 10 ms, compared to 2 ms to 3 ms in Set 1 conditions. As indicated in Fig. 9, the area near #3 is predominantly in the liquid R134a refrigerant, hence the temperature at this point remains at the inflow temperature of 288 K, with occasional brief increases in pressure when nearby cavities collapse.

**Fig. 17:**
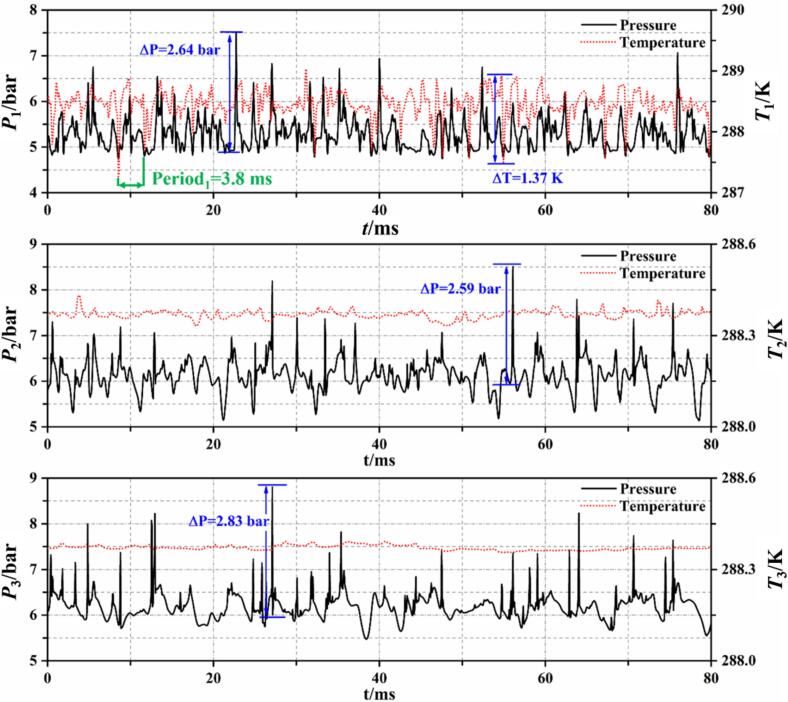
Wall temperature and pressure fluctuations at Set 3 condition.

As shown in Fig. 11, under Set 3 conditions, cavities mainly merge into cavitation clouds in the section of the throat. The continuous generation and collapse of bubbles passing through #1 cause temperature fluctuations at this point around the inflow temperature (see Fig. 17). The frequency of these temperature fluctuations is shorter compared to the previous two conditions. For #2 and #3, located in the downstream diffuser section of the VT, they were largely unaffected by upstream cavitation, with the measured temperature and pressure showing minimal fluctuations.

Incorporating the study of cavitation-induced fluctuations in wall temperature and pressure, it is evident that during intense cavitation, the cavitating flow of R134a refrigerant does not exhibit significant periodicity. As the cavitation number increases, the flow transitions to quasi-periodicity. Influenced by thermal effects, the interaction between vortices and cavitation becomes more pronounced, making the cavitation process in thermosensitive fluids more complex and unstable.

## 5 Conclusions

In the present work, the transient cavitating flow of R134a refrigerant in a VT under varying cavitation numbers (σ= 1.522, 1.807 and 2.274) was investigated. A numerical simulation model based on S-S cavitation model was conducted, and the feasibility of the model was validated through experiments. This study revealed the spatiotemporal evolution of cavitation clouds and the interaction of thermodynamic effects and vortices with cavitation. The main conclusions can be drawn:

(1) The bubble number density *n_b_* in the cavitation model for R134a refrigerant is lower than the number density of 10^13^, which is typically applicable for water. The simulation results for pressure and temperature distribution inside the VT align well with experimental data within the studied temperature range (278 K ∼ 288 K). The predicted error in cavitation cloud length is 5.1 %;
(2) Employing LES simulation and considering the thermodynamic effects of R134a refrigerant, the vorticity transport equation revealed two shedding patterns of cavitation clouds. One pattern is triggered by a combination of reentrant flow from wall and the centrifugal effect of vortex, leading to an increase in vorticity. The other is caused by the interaction of the central jet in the mainstream and the reverse jet produced by collapsing bubbles;
(3) The thermodynamic effects inhibited the occurrence of refrigerant cavitation. Under the combined influence of thermodynamic effects and vortex, partial shedding of blunt body cavitation clouds occurred, resulting in an unstable cavitation boundary. In the isothermal model, cavitation degree was over-predicted, with temperature differences in the core of cavitation clouds reaching up to 50 K. In contrast, the non-isothermal model, which considered thermodynamic effects, showed a maximum temperature drop of only 2.5 K;
(4) Although temperature and pressure changes within the cavitation region occur synchronously, with the cavity collapse, the variation in temperature slightly lags the pressure. The speed of instantaneous pressure wave propagation during bubble collapse is greater than the convective heat transfer rate, which is in turn faster than conductive heat transfer rate. This leads to a lag of 0.1 ms to 0.3 ms in local temperature responses to pressure changes.

## CRediT authorship contribution statement

**Beile Zhang:** Writing – review & editing, Writing – original draft, Visualization, Software. **Ze Zhang:** Writing – review & editing, Data curation, Conceptualization. **Xufeng Fang:** Methodology, Investigation, Conceptualization. **Rong Xue:** Funding acquisition. **Shuangtao Chen:** Project administration. **Yu Hou:** Supervision, Resources.

## Declaration of competing interest

The authors declare that they have no known competing financial interests or personal relationships that could have appeared to influence the work reported in this paper.
